# Prediction Quality of Glucose Trend Indicators in Two Continuous Tissue Glucose Monitoring SystemsParts of these data were previously presented at the 53rd Annual Meeting of the European Association for the Study of Diabetes, September 11–15, 2017, Lisbon, Portugal.

**DOI:** 10.1089/dia.2018.0112

**Published:** 2018-08-01

**Authors:** Guido Freckmann, Manuela Link, Antje Westhoff, Ulrike Kamecke, Stefan Pleus, Cornelia Haug

**Affiliations:** Institut für Diabetes-Technologie, Forschungs- und Entwicklungsgesellschaft mbH an der Universität Ulm, Ulm, Germany.

**Keywords:** Blood glucose monitoring replacement, Continuous glucose monitoring, Nonadjunctive use, Trend arrow, Trend indicator.

## Abstract

***Background:*** Continuous interstitial glucose monitoring (CGM) systems often provide glucose trend indicators (e.g., arrows) in addition to current glucose values. These indicators are recommended to be used in therapeutic decisions, because they are ascribed predictive qualities by CGM system manufacturers and expert committees. This study assessed how reliably trend indicators match future glucose change, because such information is missing.

***Methods:*** In a clinical trial, two different CGM systems were used by 20 participants, with two sensors of each system per patient. Participants used the systems for 14 days with three study site visits (48 h each). During study site visits, glucose trend indicators, as displayed by the CGM systems, were recorded at least once per hour during daytime and once at night in a diary. In addition, CGM data were downloaded from the devices.

Trend indicators were compared with glucose change calculated from CGM data >30 min after recording the trend indicator.

***Results:*** Approximately 60% of trend indicators matched the glucose change calculated from CGM data. More than 10% of trend indicators differed by at least two trend indicator categories. Focusing on trend indicators recorded around carbohydrate (CHO) intake and insulin deliveries resulted in approximately half of trend indicators matching the calculated glucose change.

***Conclusions:*** Trend indicators do not always match future glucose change, especially within the first few hours after CHO intake and insulin deliveries. Manufacturers' labeling and recommendations should reflect this, so that CGM users can make informed decisions.

## Introduction

Continuous interstitial glucose monitoring (CGM) is used increasingly often in therapy of diabetes mellitus. In contrast to blood glucose (BG) monitoring, CGM provides a more comprehensive picture of the user's glucose concentrations, because current glucose concentrations are displayed much more frequently, for example, up to every 5 min in real-time CGM or up to every 1 min in intermittently scanned/viewed CGM. Often, CGM systems also display a graph of recent glucose readings, and they provide trend indicators. However, most CGM systems measure interstitial tissue glucose (TG) concentrations, which are known to differ from BG concentrations.^1,2^

The trend indicators of CGM systems may impact a diabetes patient's therapy under specific circumstances. In sensor-augmented pump therapy, for example, the insulin pump may suspend insulin delivery if the predicted glucose concentration crosses a preset hypoglycemia threshold. In artificial pancreas closed-loop systems, the control algorithm may increase or decrease insulin delivery based on rising or falling glucose concentrations. Even in the absence of such systems, patients may adjust insulin dosing based on trend indicators.^3–5^

In the European Union, two TG monitoring systems are intended for nonadjunct use, that is, to replace BG monitoring in many situations: the real-time CGM system Dexcom G5^®^ Mobile (DG5; Dexcom, Inc., San Diego, CA) and the so-called “flash glucose monitoring” system FreeStyle Libre (FL; Abbott Diabetes Care, Alameda, CA). The FL system does not provide real-time data, but instead relies on intermittent scanning of the sensor, upon which current TG information is displayed. Both manufacturers describe in their product labeling that trend indicators tell the user where glucose “is heading.”^6,7^ For DG5, the labeling includes information on how trend indicators should be taken into account when therapeutic decisions are based on TG readings. For FL, however, the information provided regarding use of trend indicators seems to be country specific, because instructions for use differ in that regard.

In two landmark trials, participants were instructed to consider trend indicators when delivering premeal insulin doses by adjusting doses by up to ±20%.^8,9^ In recently published survey data, diabetes patients acknowledged that they would increase premeal insulin doses by up to 400% in case of rapidly rising glucose concentrations or possibly skip premeal insulin doses in case of rapidly decreasing glucose concentrations.^3^ Recent recommendations also suggest that circumstances like meals should be taken into account when using trend indicators for insulin dose adjustments.^5,10^

Considering the potential impact of trend indicators on diabetes therapy, this evaluation aimed at assessing how well trend indicators in DG5 and FL match future glucose changes calculated from TG and BG measurement data.

## Methods

This investigator-initiated, open-label, single-center, single-arm, interventional trial was conducted between March 2016 and October 2016 at the Institut für Diabetes-Technologie Forschungs- und Entwicklungsgesellschaft mbH an der Universität Ulm, Ulm, Germany in compliance with Good Clinical Practice provisions, and local laws and regulations. The study protocol was approved by the responsible independent Ethics Committee, and the trial was exempted from regulatory approval by the competent authority. The trial was registered in the German Clinical Trial Register (“Deutsches Register Klinischer Studien,” DRKS) with the registration number DRKS00011920, an approved Primary Register in the WHO International Clinical Trials Registry Platform.

### Participants

Potential participants were selected from a subject database at the study site. After providing informed consent, participants were screened in a physical examination by a study physician in which eligibility criteria were checked. Participants had to be adult (at least 18 years of age), and they had to have type 1 diabetes using either multiple daily injections or continuous subcutaneous insulin infusion. For participants older than 45 years of age, additional cardiovascular risk factors (besides age and having type 1 diabetes) were checked, for example, smoking, arterial hypertension, and hyperlipidemia. Participants were excluded from the trial if they exhibited more than one additional risk factor. Participants were also excluded for the following reasons: severe acute or chronic illness besides diabetes mellitus or history of any illness that, in the opinion of the investigator, might confound the results of the study or pose additional risk in applying the medical device to the patient; pregnancy or lactation period; significantly impaired awareness of hypoglycemia; severe skin abnormalities or skin diseases at the potential sensor insertion sites; mental incapacity or language barriers precluding adequate compliance with the study procedures; legal incompetence or limited legal competence; or dependency from the sponsor or the clinical investigator.

Out of 23 participants that were contacted for study participation, 20 met eligibility criteria and were enrolled in the study.

### Continuous tissue glucose monitoring systems

In this study, two systems were used, the DG5 and FL systems. The hand-held DG5 receiver displayed TG and trend information in real time, sent from the sensor unit through a wireless transmitter, providing new information every ∼5 min. With FL, the hand-held reader had to be held in close proximity of the sensor unit, that is, the sensor had to be “scanned,” to obtain current TG and trend information. FL displayed trend information, the “scanned” TG result, and a continuous TG trace. Continuous TG data were recorded by FL every 15 min. The number of trend indicators as well as the corresponding TG rates of change differed between the two systems as shown in Table 1.

**Table 1.** Definition of Trend Indicators (arrows) displayed by Dexcom G5 and FreeStyle Libre as Defined in the Respective User Manuals (DG5: LBL013328 Rev 001; FL: ART28687-102 Rev. A 04/14)

	*Associated glucose rate of change*	
*Trend indicator(arrow)*	*DG5*	*FL*
⬆⬆	Glucose rapidly rising >3 mg/dL each minute or >45 mg/dL in 15 min.	n.a.
⬆	Glucose rising 2–3 mg/dL each minute or up to 45 mg/dL in 15 min.	Glucose is rising quickly [>2 mg/(dL·min)]
⬈	Glucose slowly rising 1–2 mg/dL each minute or up to 30 mg/dL in 15 min.	Glucose is rising [between 1 and 2 mg/(dL·min)]
➔	Glucose is steady. Not increasing/decreasing >1 mg/(dL·min) or up to 15 mg/dL in 15 min.	Glucose is changing slowly [<1 mg/(dL·min)]
⬊	Glucose is slowly falling 1–2 mg/dL each minute or up to 30 mg/dL in 15 min.	Glucose is falling [between 1 and 2 mg/(dL·min)]
⬇	Glucose is falling 2–3 mg/dL each minute or up to 45 mg/dL in 15 min.	Glucose is falling quickly [>2 mg/(dL·min)]
⬇⬇	Glucose is rapidly falling >3 mg/dL each minute or >45 mg/dL in 15 min.	n.a.

DG5, Dexcom G5; FL, FreeStyle Libre; n.a., not applicable.

DG5 was calibrated as per the manufacturer's labeling (every 12 h), whereas FL was factory calibrated.

According to the labeling of the study devices, DG5 is designed to be used for treatment decisions as long as symptoms match sensor glucose readings and if the following information is available and used: (1) most recent sensor glucose reading, (2) glucose reading graph (at least for the recent 15 min), (3) trend arrow, and (4) alarms/alerts. FL is also intended as replacement for BG monitoring, with BG measurements being necessary when glucose concentrations are rapidly changing [>2 mg/(dL·min)], when sensor readings report hypoglycemia or impending hypoglycemia, if symptoms do not match sensor readings, or if the user suspects sensor readings to be incorrect.

For both systems, device labeling includes further information about the general use of the systems, for example, regarding interfering substances.

### Study design

**FIG. 1.**
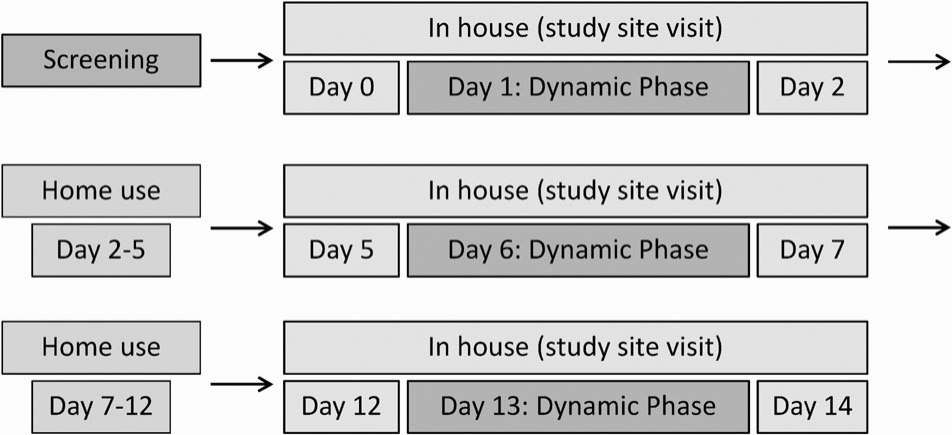
Study timeline. Trend indicators were recorded with every BG measurement during in-house phases. BG measurements were performed at 0300 and once per hour between 0600 and 2400 during in-house phases, as well as every 15 min during dynamic phases (0800–1300 at days 1, 6, and 13). BG, blood glucose.

Within 6 weeks of the screening visit, the 14-day experiment phase started. During this experiment phase, participants visited the study site three times for ∼48 h each. Between the three study site visits, participants were at home for 72 and 120 h, respectively. The study timeline is shown in Figure 1.

On the first day of the first study site visit (day 0), a study physician applied two sensors of each of the two systems to the participants: one FL sensor was worn on each upper arm, the two DG5 sensors were placed on the left and right side of the abdomen. Because DG5 is indicated for 7 days of use, the sensors were routinely replaced on day 7.

While at the study site, participants performed BG measurements at least once per hour between 0600 and 2400, and once at night at ∼0300. In parallel to each BG measurement, FL was scanned and the current glucose trend indicators from all four sensors (two sensors for each of two systems) were recorded in a diary. Manual documentation was performed because data download from the TG systems did not include glucose trend indicators. At home, participants did not record glucose trend indicators.

Rapidly changing glucose concentrations with BG values in the hyperglycemic and hypoglycemic ranges were induced on 1 day of each study site visit (“dynamic phases”). Participants ate a high glycemic index breakfast and delayed and increased their corresponding insulin bolus dose. From 30 min before breakfast until 5 h after breakfast, BG was monitored closely with one measurement every 15 min. Participants were closely supervised by study staff and a study physician until BG returned to normoglycemic values. If BG concentrations dropped <55 mg/dL or if hypoglycemia symptoms were reported by participants, 18 grams of carbohydrates (CHOs) were administered. An increased frequency of BG measurements was sustained until BG concentrations were outside the hypoglycemic range.

### Data analysis

Glucose trend indicators as documented in the participants' diaries were compared with glucose changes calculated from linearly interpolated TG readings. For each of the four simultaneously worn sensors (two for DG5, two for FL), the recorded trend indicator was compared with the respective sensor's glucose readings.

For each trend indicator record, TG readings obtained from data downloads were used to calculate TG change in the 30 min after the trend indicator was recorded. For FL, only continuously stored data were used. In this analysis, TG readings were linearly interpolated at the time the trend indicator was recorded, that is, at times of BG measurements and 30 min afterward. Linear interpolation was performed so that both systems could be analyzed in the same manner, despite DG5 recording TG values three times as often as FL.

The calculated TG change was attributed to a trend indicator based on the information provided in the respective user manuals, given in Table 1. For example, if the calculated TG change was +36 mg/dL after 30 min, or +1.2 mg/(dL·min), it was categorized as one arrow pointing diagonally upward (see also Supplementary Fig. S1; Supplementary Data are available online at www.liebertpub.com/dia). The calculated trend indicator and the recorded trend indicator were compared. Contingency tables for these comparisons and descriptive statistics for differences in trend indicator categories were calculated (see Supplementary Table S1). In addition, data were stratified by study site visit to estimate influence of sensor wear. Missing data, for example, if one of the systems did not provide a trend indicator, were not replaced. A similar analysis comparing trend indicators to glucose changes calculated from BG values was also performed (see Supplementary Table S2; Supplementary Fig. S2).

In an additional analysis, trend indicators whose 30-min observation window overlaps with CHO intake or insulin delivery were analyzed. The rationale behind this exclusion was that patients should know that trend indicators cannot foresee CHO intake or insulin delivery, so that prediction quality is likely limited if the 30-min observation window described above overlaps with CHO intake or the first 2 h afterward. The complementary analysis of trend indicators recorded >30 min before CHO intake or insulin delivery or >120 min afterward was also performed.

## Results

### Study population

A total of 20 participants (8 female, 12 male; all Caucasian) were enrolled in the study. Participants were 21–64 years old (mean ± standard deviation: 39.0 ± 13.2 years) and their average body mass index was 26.3 ± 3.9 kg/m^2^, ranging between 20.5 and 37.5 kg/m^2^. All participants had type 1 diabetes with either continuous subcutaneous insulin infusion (70%) or multiple daily injections (30%) as their therapy regimen. Diabetes was diagnosed an average of 21.2 ± 10.8 years (range: 1–45 years) before enrollment. At the screening visit, glycated hemoglobin (HbA_1c_) level ranged from 5.7% to 9.6% (38.8–81.4 mmol/mol), with average HbA_1c_ of 7.2% ± 1.1% (55.6 ± 11.6 mmol/mol).

### Indicated TG trend versus calculated TG trend converted to trend category based on all available data

**FIG. 2.**
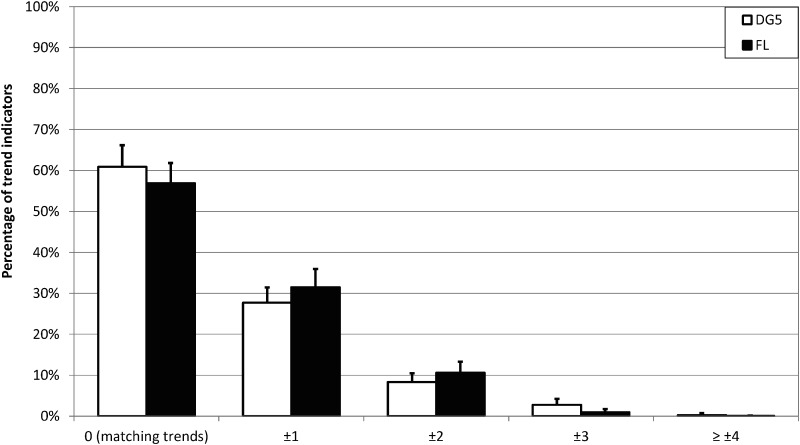
Absolute differences in categories between indicated TG trend and trend calculated from TG readings over the following 30 min for DG5 and FL. Mean value and standard deviation for participant-specific results are displayed. DG5, Dexcom G5; FL, FreeStyle Libre.

For the comparison of indicated TG trends versus trends calculated from TG readings based on all available data (*n* = 6575 for DG5, *n* = 5957 for FL; grouped by *n* = 20 participants), 60.9% and 56.9% of DG5 and FL trend indicators, respectively, matched the TG change over the following 30 min (see Fig. 2; Supplementary Table S1). For the two systems, in 11.4% (DG5) and 11.6% (FL) of cases the trends were different by at least two categories. When data were stratified by study site visit, a slightly smaller number of trend indicators matched the TG change during the first study site visit (56.3% for DG5, 53.8% for FL), and the number of cases in which trends differed by at least two categories was slightly larger (14.4% for DG5, 13.8% for FL) than during the following visits (see Table 2).

**Table 2.** Indicated Tissue Glucose (TG) Trend Versus Trend Calculated from TG Readings Over the Following 30 Min for Dexcom G5 and FreeStyle Libre, Stratified by Study Site Visit

	*Visit 1*		*Visit 2*		*Visit 3*	
*Categories difference*	*DG5*	*FL*	*DG5*	*FL*	*DG5*	*FL*
0 (matching), % (*n*)	56.3 (1206)	53.8 (1123)	63.7 (1404)	58.3 (1218)	62.0 (1381)	59.1 (1053)
±1, % (*n*)	29.4 (629)	32.4 (675)	26.5 (585)	31.4 (656)	27.7 (616)	30.5 (544)
≥ ±2, % (*n*)	14.4 (308)	13.8 (288)	9.8 (216)	10.3 (215)	10.3 (230)	10.4 (185)
Total, *n*	2143	2086	2205	2089	2227	1782

Visit 1: days 0–2, sensor insertion for both systems on day 0. Visit 2: days 5–7, replacement of DG5 sensor on day 7 before participants left. Visit 3: days 12–14, sensor removal for both systems on day 14.

Comparison of trends indicated by the two sensors of the same CGM system worn in parallel by the same participant showed that the majority of trend indicators matched (82.4% for DG5, 80.7% for FL), and only few trend indicators (0.8% for DG5, 0.6% for FL) were different by at least two categories (see Supplementary Table S3).

In a similar analysis based on a comparison of indicated TG trends versus trends calculated from BG readings, comparable results were found (see Supplementary Table S2; Supplementary Fig. S2).

### Additional analysis of indicated TG trend versus calculated TG trend regarding times of CHO intake or insulin delivery

Trend indicators were recorded <30 min before or <120 min after CHO intake or insulin delivery in 4629 cases for DG5 and in 4093 cases for FL. This corresponds to 70.4% and 68.7% of all recorded DG5 and FL trend indicators, respectively. Results were again grouped by *n* = 20 participants.

**FIG. 3.**
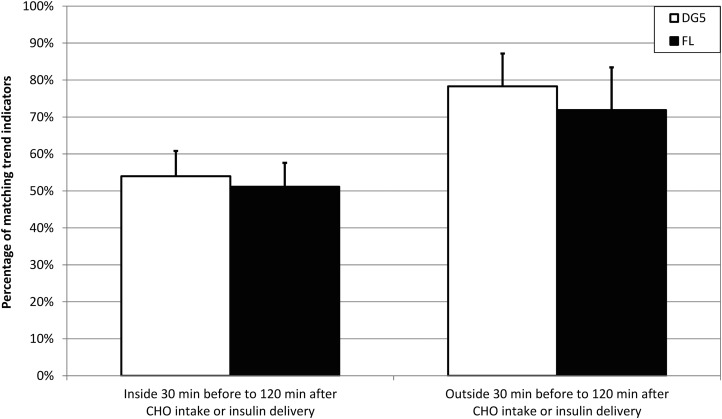
Percentage of trend indicators matching trends calculated from TG readings over the following 30 min for DG5 and FL. Left: trend indicators recorded <30 min before or <120 min after CHO intake or insulin delivery. Mean value and standard deviation for participant-specific results are displayed. Right: trend indicators recorded >30 min before or at least 120 min after CHO intake or insulin delivery. CHO, carbohydrate.

Outside of the specified time windows, 78.3% of DG5 trends and 71.9% of FL trends matched trends calculated from TG readings, whereas inside these time windows, this was the case for 54.0% and 51.1% of DG5 and FL trend indicators, respectively (see Fig. 3).

## Discussion

In this evaluation, the prediction quality of TG trend indicators as displayed by CGM systems was assessed by comparing the TG trend indicator to subsequent glucose changes that were retrospectively calculated from linearly interpolated TG readings.

The presumption that TG trend indicators have predictive qualities at all is based on the following aspects: First, the instructions for use for DG5 and FL (and, possibly, other CGM systems) provided by the respective manufacturer indicate that trend arrows show where glucose “is heading.”^6,7^ Second, insulin pump systems incorporating predictive low glucose management have to estimate impending hypoglycemia, and trend arrows would be the logical basis for such estimations. Many closed-loop glucose control systems also have some kind of glucose prediction algorithms, although they may not necessarily use trend indicators provided by the CGM systems.

The approach presented in this study is comparably simple and can easily be adapted to other timeframes, as long as glucose readings are available often enough. Calculations were performed as described to assess whether trend indicators match glucose change calculated from TG readings. However, CHO intake, bolus insulin delivery, as well as physical exercise, which were not performed in this study, should be considered in such assessments.

TG change was calculated based on TG readings over the following 30 min after trend arrows were recorded. This interval was chosen in reference to the functionality of the MiniMed^®^ 640G SmartGuard^®^ (Medtronic MiniMed, Northridge, CA) insulin pump (which was not investigated in this evaluation) that incorporates a predictive low glucose management based on 30-min estimates of glucose changes.^11^

In this analysis of trend indicators versus TG trends retrospectively calculated from TG readings, ∼60% of TG trends matched the calculated TG change over the following 30 min. Slightly >10% of trends differed by at least two categories. The majority of “stable” glucose trend indicators [i.e., less than ±1 mg/(dL·min) rate of change] matched the calculated TG change, whereas the majority of rising and falling glucose trend indicators suggested higher rates of change than TG change calculated from downloaded data. For falling glucose levels, this could be seen as a conservative approach, because if a patient's actual TG values were falling slower than indicated, hypoglycemia would result less often than when TG values were falling faster than indicated. For rising glucose levels, however, this property of the investigated CGM systems could possibly lead to increased risk of hypoglycemia. According to survey data from Pettus and Edelman,^3^ patients would more than double their glucose correction doses if a single or double upward trend arrow were displayed accompanied by a hyperglycemic sensor reading. Such actions could possibly induce hypoglycemia, if the actual TG change is largely overestimated.

In the additional analysis excluding the 30 min before and 120 min after CHO intake and insulin delivery, considerably more trend indicators matched the calculated TG change (∼75%). When focusing on these times of CHO intake or insulin delivery, approximately half of trend indicators did not match the calculated TG change.

During the first study site visit, slightly larger differences in trend categories were found than during the other two visits. This could be influenced by the two CGM systems exhibiting reduced measurement performance in the first few hours after sensor insertion, a phenomenon that is often observed with CGM systems.^12,13^

Recently, multiple recommendations on using trend arrows in diabetes therapy have been published.^4,5,10^ This evaluation's results suggest that trend arrows should only be used carefully within the first few hours after CHO intake and insulin deliveries, even outside of meals. Patients should be taught the necessary skills to interpret previous glucose readings and put them into the appropriate context regarding previous CHO intake, insulin delivery, and exercise.

The clinical relevance of this evaluation's findings could be limited, for example, if patients adapt their therapy regimen based on unsatisfactory experiences: If undesirable glucose levels were induced by a mismatch between trend indicators and actual TG or BG change, patients could learn from these experiences and perform more appropriate reactions to trend indicators in the future. In insulin pump systems with predictive low glucose management, however, hypoglycemia or hyperglycemia could be inadvertently induced because suspension or resumption of insulin delivery may be inadequate. It should be pointed out that the two investigated systems did not offer such management options at the time of the study, so that this hypothesis could not be tested with the systems at hand.

Studies about analytical performance of CGM systems show discrepancies between TG and BG readings. This is acknowledged by manufacturers, who typically provide performance data in the systems' instructions for use. Although at least some manufacturers suggest that trend indicators have some predictive aspect, performance data are missing. Patients might therefore mistakenly think that trend indicators are appropriate all of the time. The evaluation presented here suggests that this is not the case, because trend indicators do not necessarily match future TG change. It would be helpful if potential discrepancies between trend indicators and future glucose change were part of the systems' labeling.

Information about point accuracy and, to a smaller extent, rate-of-change accuracy (sometimes also called trend accuracy) of CGM systems is available publicly.^14–18^ Information regarding the prediction quality of trend indicators, however, is scarce, despite its importance to CGM-based diabetes therapy. Further studies are required, focusing on other CGM systems as well, to provide a more comprehensive picture. Although the two investigated CGM systems showed qualitatively similar results, other systems might not. If different CGM systems showed systematically different behavior, adjusting insulin doses based on trend indicators might be further impacted.

## Supplementary Material

Supplemental data
