# Supplementary material for: Prediction Quality of Glucose Trend Indicators in Two Continuous Tissue Glucose Monitoring Systems
Source: Diabetes Technol Ther. 2018 Aug 1;20(8):550–6. doi: 10.1089/dia.2018.0112 (PMC6080115; doi:10.1089/dia.2018.0112)
Supplement: Supplemental data [file Supp_Data.pdf]

Supplementary Data

Graphical Presentation of Calculation Procedures

See Supplementary Figure S1.

Analysis of Indicated Tissue Glucose Trend Versus Calculated Tissue Glucose Trend

Supplementary Table S1 shows the occurrence of pairs of indicated trends (columns) and trends calculated from tissue glucose (TG) readings over the following 30 min. Calculated trends were determined by calculating the difference between the TG readings, divided by 30 min. The resulting TG rate of change was then categorized based on manufacturer's labeling to a corresponding trend indicator (see also Table 1 in the article).

Analysis of Indicated TG Trend Versus Calculated Blood Glucose Trend

For each trend indicator record, blood glucose (BG) readings obtained from data downloads were used to calculate BG change between the BG measurement at the time the trend indicator was recorded and a BG measurement after ~60–75 min. Calculation was only performed if a BG measurement was obtained within ~60–75 min after the trend indicator was recorded. If multiple BG measurements were obtained in this timeframe, the first of these BG measurements was used. BG data were not interpolated, because during daytime outside of dynamic phases only one BG measurement per hour was performed, so that linear interpolation would not have adequately reflected actual BG change. Again, calculated trends were determined by categorizing the BG rate of change based on the manufacturer's labeling.

For the comparison of indicated TG trend versus trend calculated from BG readings [$n = 3794$ for Dexcom G5 (DG5), $n = 3453$ for FreeStyle Libre (FL)], 60.9% and 58.3% of DG5 and FL trend indicators, respectively, matched the BG change over the following 60–75 min. In 11.6% and 12.5% of cases, respectively, the trends were different by at least two categories (Supplementary Table S2; Supplementary Fig. S2).

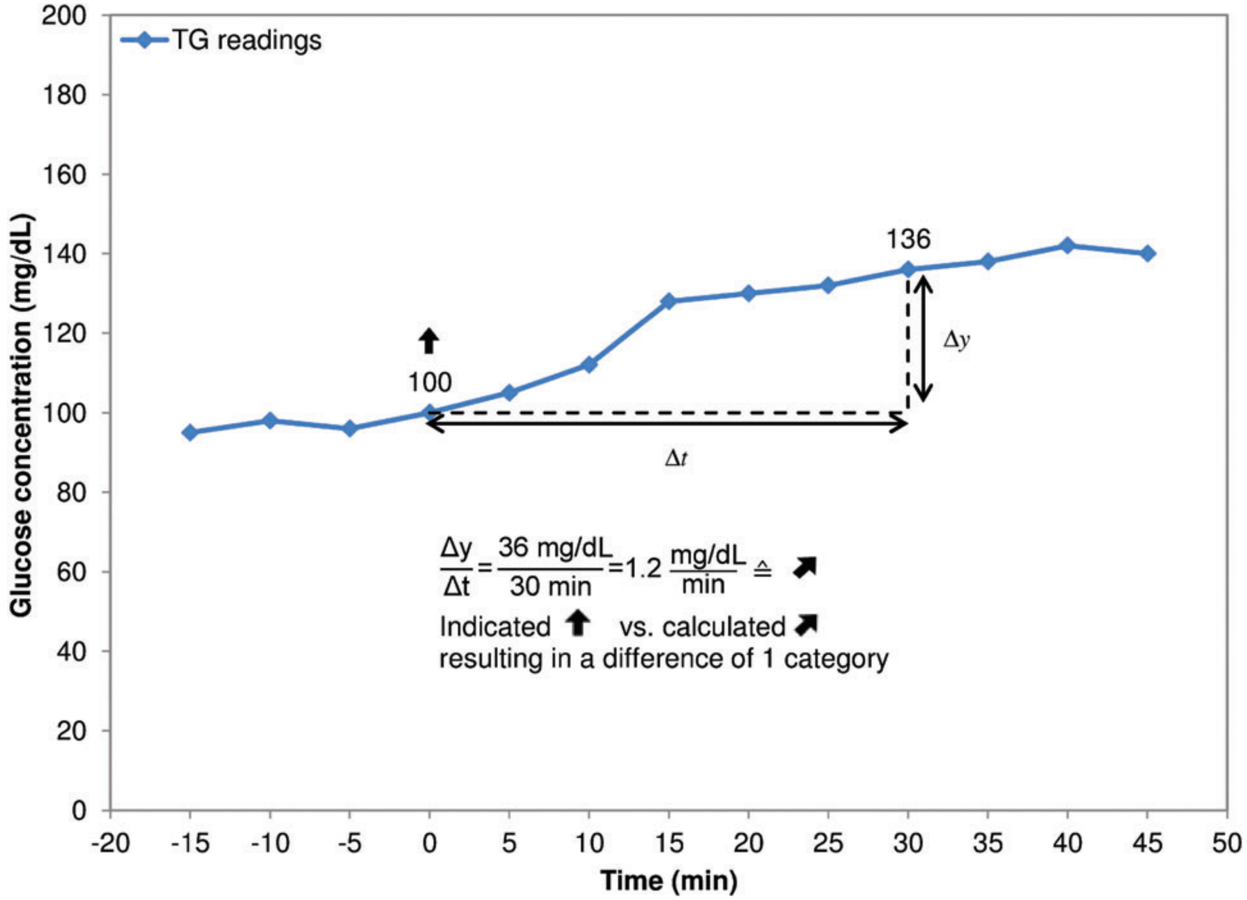

SUPPLEMENTARY FIG. S1. Calculation of trend categories derived from linearly interpolated TG readings. The TG change over the following 30 min after recording a trend indicator was calculated. This change was converted to a glucose rate of change and subsequently attributed a trend indicator based on the respective user manuals. TG, tissue glucose.

Supplementary Table S1. Indicated Tissue Glucose (TG) Trend Versus Trend Calculated from TG Readings Over the Following 30 Min for Dexcom G5 and FreeStyle Libre

		Indicated TG trend						
		⬇⬇	⬇	⬊	➡	⬈	⬆	⬆⬆
**Trend calculated from TG readings (DG5)**	⬇⬇	0.08%	0.06%	0.05%	0.05%	0.02%	0.02%	0.00%
	⬇	0.11%	0.49%	0.85%	0.65%	0.11%	0.00%	0.03%
	⬊	0.20%	1.75%	5.00%	4.76%	0.62%	0.26%	0.15%
	➡	0.11%	0.94%	7.42%	53.43%	6.74%	2.33%	0.93%
	⬈	0.02%	0.11%	0.71%	4.44%	1.23%	0.79%	0.90%
	⬆	0.00%	0.02%	0.15%	1.69%	0.33%	0.30%	0.44%
	⬆⬆	0.00%	0.00%	0.02%	1.10%	0.32%	0.14%	0.17%
**Trend calculated from TG readings (FL)**	⬇	n.a.	1.33%	0.47%	0.60%	0.07%	0.03%	n.a.
	⬊	n.a.	3.07%	3.24%	4.52%	0.64%	0.57%	n.a.
	➡	n.a.	1.80%	9.30%	50.60%	7.42%	3.56%	n.a.
	⬈	n.a.	0.10%	0.84%	4.92%	0.74%	1.29%	n.a.
	⬆	n.a.	0.02%	0.18%	3.14%	0.49%	1.07%	n.a.

For definition of trend indicators (arrows), see Table 1. Green, indicated trend matches trend calculated from TG readings. Red, indicated trend shows different direction than glucose change calculated from TG readings.

DG5, Dexcom G5; FL, FreeStyle Libre; n.a., not applicable; TG, tissue glucose.

Supplementary Table S2. Indicated Tissue Glucose Trend Versus Trend Calculated from Blood Glucose Readings Over the Following 60–75 Min for Dexcom G5 (upper part of the table) and FreeStyle Libre (lower part of the table)

		Indicated TG trend						
		⬇⬇	⬇	⬊	➡	⬈	⬆	⬆⬆
**Trend calculated from BG readings**	⬇⬇	0.00%	0.00%	0.00%	0.00%	0.00%	0.00%	0.00%
	⬇	0.08%	0.16%	0.26%	0.26%	0.32%	0.11%	0.05%
	⬊	0.03%	0.50%	1.92%	3.58%	0.87%	0.69%	0.32%
	➡	0.18%	1.61%	7.09%	58.38%	9.09%	2.58%	0.66%
	⬈	0.03%	0.13%	0.90%	6.54%	0.47%	0.29%	0.11%
	⬆	0.00%	0.00%	0.05%	2.50%	0.03%	0.00%	0.00%
	⬆⬆	0.00%	0.00%	0.00%	0.21%	0.00%	0.00%	0.00%
**Trend calculated from BG readings**	⬇	n.a.	0.23%	0.14%	0.35%	0.14%	0.17%	n.a.
	⬊	n.a.	1.19%	1.48%	3.50%	0.93%	0.96%	n.a.
	➡	n.a.	2.32%	7.59%	56.21%	9.50%	3.79%	n.a.
	⬈	n.a.	0.29%	0.90%	6.86%	0.35%	0.29%	n.a.
	⬆	n.a.	0.00%	0.12%	2.58%	0.09%	0.03%	n.a.

For definition of trend indicators (arrows), see Table 1. Green, indicated trend matches trend calculated from BG readings. Red, indicated trend shows different direction than glucose change calculated from BG readings.

BG, blood glucose.

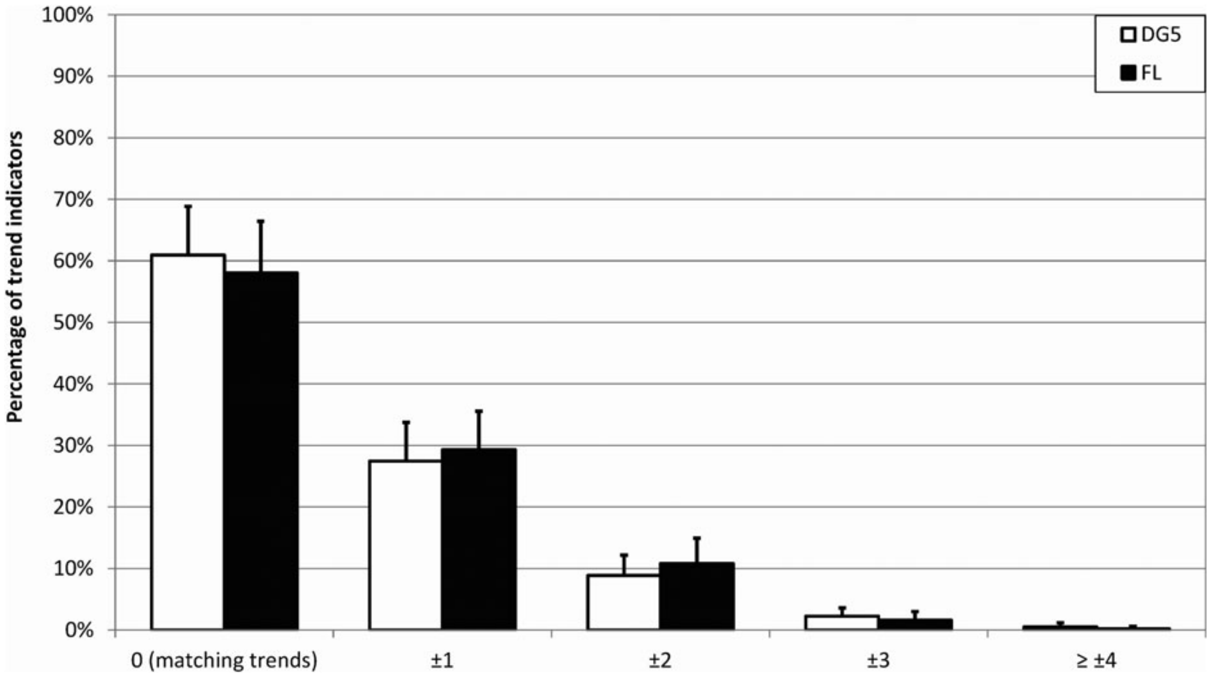

SUPPLEMENTARY FIG. S2. Absolute differences in categories between indicated TG trend and trend calculated from BG readings over the following 60–75 min for DG5 and FL. BG, blood glucose; DG5, Dexcom G5; FL, FreeStyle Libre.

The comparison with BG change could be improved by performing high-frequency BG measurements throughout the day. This would allow an assessment of short-term prediction quality of these trend indicators. Because in the study on which the evaluation presented here is based, high-frequency BG measurements were only performed at times of rapidly changing glucose concentrations, such an assessment would have been unbalanced and was, therefore, not performed.

SUPPLEMENTARY TABLE S3. DIRECT COMPARISON OF TREND INDICATORS AS INDICATED BY THE TWO SENSORS OF THE SAME CONTINUOUS TISSUE GLUCOSE MONITORING SYSTEM (DEXCOM G5 AND FREESTYLE LIBRE) IN A PARTICIPANT

Categories difference	*DG5*	*FL*
0 (matching), % (*n*)	82.4 (2728)	80.7 (2180)
±1, % (*n*)	16.8 (555)	18.7 (504)
≥ ±2, % (*n*)	0.8 (28)	0.6 (16)
Total, *n*	3311	2700

Comparison of Trend Indicators as Indicated by the Two Sensors of the Same Continuous TG Monitoring System

In this additional analysis, the two sensors of a CGM system that were worn by the same participant were compared directly. Because each participant wore two sensors of each CGM system in parallel (i.e., at the same time), glucose concentrations and trends recorded by the two sensors of each system should not be fundamentally different (Supplementary Table S3).

The majority of trend indicators as indicated by the two sensors of the same CGM system worn by a participant matched, and only few trend indicators were different by at least two categories.
